# Chebyshev centers and radii for sets induced by quadratic matrix inequalities

**DOI:** 10.1007/s00498-025-00424-w

**Published:** 2025-10-23

**Authors:** Amir Shakouri, Henk J. van Waarde, M. Kanat Camlibel

**Affiliations:** Bernoulli Institute for Mathematics, Computer Science and Artificial Intelligence, University of Groningen, Nijenborgh 9, 9747AG Groningen, The Netherlands

**Keywords:** Quadratic matrix inequality, Chebyshev center, Chebyshev radius, Unitarily invariant norm, Data-driven control, System identification

## Abstract

This paper studies sets of matrices induced by quadratic inequalities. In particular, the center and radius of a smallest ball containing the set, called a *Chebyshev center* and the *Chebyshev radius*, are studied. In addition, this work studies the *diameter* of the set, which is the farthest distance between any two elements of the set. Closed-form solutions are provided for a Chebyshev center, the Chebyshev radius, and the diameter of sets induced by quadratic matrix inequalities (QMIs) with respect to arbitrary unitarily invariant norms. Examples of these norms include the Frobenius norm, spectral norm, nuclear norm, Schatten *p*-norms, and Ky Fan *k*-norms. In addition, closed-form solutions are presented for the radius of the largest ball *within* a QMI-induced set. Finally, the paper discusses applications of the presented results in data-driven modeling and control.

## Introduction

Quadratic matrix inequalities (QMIs) are ubiquitous in systems and control theory. Prominent examples are the Lyapunov and the algebraic Riccati inequality. The study of QMI-induced sets is classically motivated by the design of optimal and robust controllers [1–6], and recently, has seen a renewed interest due to its applications in data-driven modeling and control [7–10]. Latest studies on certain types of QMI-induced sets have led to the development of matrix versions of several well-known results such as Yakubovich’s S-lemma [7, 11] and Finsler’s lemma [12, 13]. Research on QMI-induced sets is also relevant for matrix regression and system identification where the noise model may be given by quadratic inequalities.

Given a compact set of matrices and a norm, the center and radius of a smallest ball containing the set are referred to as a *Chebyshev center* and the *Chebyshev radius*, respectively. In addition, the farthest distance between two elements within the set is referred to as the *diameter* of the set. Such notions have applications in the approximation of matrices within QMI-induced sets. For instance, in the context of data-driven modeling of dynamical systems, given that the system matrices are unknown but belong to a QMI-induced set obtained from noisy data [7], a Chebyshev center is an estimation for the *true* system matrices, and the Chebyshev radius is the worst-case error of such an estimation. Since a Chebyshev center is a point that has the least worst-case error, it is also called a *best worst-case* estimation [14]. In this case, one may consider a Chebyshev center as the nominal system and implement tools from robust control theory to design controllers that stabilize all systems in a ball around the nominal system. The reader can refer to [15] and the references therein for more details on Chebyshev centers and their applications in other fields.

The complexity of finding a Chebyshev center and the Chebyshev radius depends on the characteristics of the set and the given norm. For instance, if the set is an ellipsoid within a Euclidean space, equipped with the Euclidean norm, then the Chebyshev center is unique and is simply the center of the ellipsoid with the Chebyshev radius being the length of the largest semi-axis. However, for an intersection of ellipsoids, there are in general no closed-form solutions and existing algorithms only converge to a proximity of the Chebyshev center [14, 16]. The effect of the given norm on the computational complexity of finding the Chebyshev radius is evident as a norm can itself be NP-hard to compute (such as matrix *p*-norms when $p\ne 1,2,\infty$ [17]). To find a Chebyshev center, there are a few nontrivial cases where a polynomial time algorithm exists for specific norms. For instance, finding a Chebyshev center for polyhedra can be reduced to a linear program [15], or, when the set is finite, one may find the smallest enclosing ball in linear time [18]. In the absence of polynomial time algorithms for finding a Chebyshev center, approximate solutions may be sought. For example, for the intersection of two ellipsoids, a relaxed Chebyshev center can be found by solving a semi-definite program [14], which is equal to the true Chebyshev center if the set is defined on the complex field. To the authors’ knowledge, a full characterization of the Chebyshev centers and radii for QMI-induced sets with respect to arbitrary unitarily invariant matrix norms is still missing in the existing literature.

In this paper, we present closed-form solutions for a Chebyshev center, the Chebyshev radius, and the diameter of a certain class of compact QMI-induced sets. In addition, we study the radius of a largest ball within a QMI-induced set, and for the cases where the set has an empty interior, we present the radius of a largest lower-dimensional ball contained in the set. All the provided solutions are with respect to arbitrary unitarily invariant matrix norms, which are expressed in terms of symmetric gauge functions [19]. Examples of such norms are the Schatten *p*-norms and the Ky Fan *k*-norms, which in particular, include spectral, nuclear, and Frobenius norms. The freedom over the choice of the norm makes it possible to approximate a QMI-induced set with respect to the norm that is most relevant to the target application.

The presented results in this paper are applied to the modeling and control of unknown linear time-invariant systems based on noisy input–output data. The noise is assumed to be unknown but belongs to a known bounded set [20–25]. In this setting, the input–output data and knowledge about the noise give rise to the *set of data-consistent systems*, which includes all systems that could have generated the data. In the case that the set of data-consistent systems is bounded, a *set-membership identification* problem seeks a solution to minimize the worst-case estimation error, that is, to find a Chebyshev center and the Chebyshev radius for the set of data-consistent systems [16, 26–28]. In this setting, the Chebyshev radius represents the identification accuracy. This accuracy needs to be computed with respect to a given norm that is most suitable for the target application. For instance, the application of the nuclear norm in system identification has been explored in [29]. The application of the spectral norm in the design of robust controllers has been studied in [30]. The relevance of the Frobenius norm in characterizing uncertainties in the system parameters has been shown in [31, 32]. Nevertheless, except for some well-known cases, such as minimizing the Frobenius norm of the data error via the least-squares problem, system identification seldom offers closed-form solutions. This motivates exploring cases for which a closed-form solution exists, which may lead to valuable information on how the input–output data affect the identification error with respect to different norms. This, in turn, can bridge the gap between direct and indirect data-driven control.

The remainder of this paper is organized as follows. Section 2 is devoted to preliminaries. Section 3 includes the main results and Sect. 4 discusses their applications in data-driven modeling and control. Finally, Sect. 5 concludes the paper.

## Preliminaries

### Notation

For $a,b\in \mathbb {Z}$ with $a\le b$, let $[a,b]{:}{=}\{x\in \mathbb {Z}:a\le x\le b\}$. Let $\mathbb {R}_+^n$ denote the set of *n*-dimensional real vectors with nonnegative entries. We define $\mathbb {R}^n_{\downarrow }{:}{=}\{x\in \mathbb {R}_+^n:x_i\ge x_{i+1},i=1,\ldots ,n\}$. For $x\in \mathbb {R}^n$ and $k\le n$ we define the vector containing the first *k* entries of *x* as $$x_{[k]}{:}{=}\begin{bmatrix} x_1&\cdots&x_k \end{bmatrix}^\top $$, and for $k>n$ we define $$x_{[k]}{:}{=}\begin{bmatrix} x^\top&0 \end{bmatrix}^\top \in \mathbb {R}^k$$. For two vectors $x,y\in \mathbb {R}^n$, we say *y* *weakly majorizes* *x*, denoted by $x\prec _w y$, if $\sum _{i=1}^k x_i\le \sum _{i=1}^k y_i$ for all $k\in [1,n]$. We define $$e_{1,n}{:}{=}\begin{bmatrix} 1&0&\cdots&0 \end{bmatrix}^\top \in \mathbb {R}^n$$, which is simply denoted by $e_1$ when its size is clear from the context.

The vector of singular values of matrix $M\in \mathbb {C}^{n\times m}$, arranged in a nonincreasing order, is denoted by $\sigma (M)\in \mathbb {R}^{\min \{n,m\}}_{\downarrow }$. Let $\sigma _*(M)$ denote the smallest nonzero singular value of $M\in \mathbb {C}^{n\times m}$ if $M\ne 0$ and $\sigma _*(M)=0$ if $M=0$. We denote the kernel of matrix $M\in \mathbb {R}^{n\times m}$ by $\ker M{:}{=}\{x\in \mathbb {R}^m:Mx=0\}$. The Moore-Penrose pseudo-inverse of a real matrix *M* is denoted by $M^\dagger$. Moreover, we denote the Hadamard (or Schur) product of two matrices $M,N\in \mathbb {R}^{n\times m}$ by $M\circ N$.

Let $\mathbb {S}^{n}$ denote the set of $n\times n$ symmetric matrices. A matrix $M\in \mathbb {S}^{n}$ is called *positive definite* (resp., *positive semi-definite*) and denoted by $M>0$ (resp., $M\ge 0$) if all its eigenvalues are positive (resp., nonnegative). By $M<0$ (resp., $M\le 0$) we imply that $-M>0$ (resp., $-M\ge 0$).

A norm $\left| \ \cdot \ \right|$ on $\mathbb {R}^{p\times q}$ is said to be *essentially strictly convex* if the unit ball in $\mathbb {R}^{p\times q}$ with respect the norm is a strictly convex set, or equivalently, $X_1,X_2\in \mathbb {R}^{p\times q}$ and $\left| X_1+X_2\right| =\left| X_1\right| +\left| X_2\right|$ imply $X_1=\alpha X_2$ for some $\alpha \ge 0$ (cf. [33, p. 106], [34, eq. 2.8]).

For a set of matrices $\mathcal {X}\subseteq \mathbb {R}^{p\times q}$ and matrices $X_0,M,N$ of appropriate sizes, we define $X_0+\mathcal {X}{:}{=}\{X_0+X:X\in \mathcal {X}\}$, $\mathcal {X}-\mathcal {X}{:}{=}\{X_1-X_2:X_1,X_2\in \mathcal {X}\}$, and $M \mathcal {X}N{:}{=}\{M XN:X\in \mathcal {X}\}$. We denote the affine hull of the set $\mathcal {X}$ by ${{\,\textrm{aff}\,}}\mathcal {X}{:}{=}\left\{ \sum _{i=1}^n\alpha _iX_i:X_i\in \mathcal {X},\alpha _i\in \mathbb {R},n\in \mathbb {N},\sum _{i=1}^n \alpha _i=1\right\}$.

### Center, radius and diameter of sets

Let $\mathcal {X}\subset \mathbb {R}^{p\times q}$ be a compact set and let $\left| \ \cdot \ \right|$ be a norm on $\mathbb {R}^{p\times q}$. We define

1 $$\begin{aligned} {{\,\textrm{diam}\,}}\mathcal {X}{:}{=}\max _{\Delta \in \mathcal {X}-\mathcal {X}} \left| \Delta \right| \end{aligned}$$

as the *diameter* of $\mathcal {X}$,

2 $$\begin{aligned} {{\,\textrm{rad}\,}}\mathcal {X}{:}{=}\min _{C\in \mathbb {R}^{p\times q}} \max _{X\in \mathcal {X}} \left| C-X\right| \end{aligned}$$

as the *Chebyshev radius* of $\mathcal {X}$, and

3 $$\begin{aligned} {{\,\textrm{cent}\,}}\mathcal {X}{:}{=}\left\{ C\in \mathbb {R}^{p\times q}:\left| C-X\right| \le {{\,\textrm{rad}\,}}\mathcal {X}\ \text {for all}\ X\in \mathcal {X}\right\} \end{aligned}$$

as the *set of Chebyshev centers*^1^^,^^2^ of $\mathcal {X}$. Due to compactness of $\mathcal {X}$, ${{\,\textrm{diam}\,}}\mathcal {X}$ and ${{\,\textrm{rad}\,}}\mathcal {X}$ are well-defined, and ${{\,\textrm{cent}\,}}\mathcal {X}$ is nonempty.

Based on the above definitions, we have the following results.

#### Proposition 1

Let $\mathcal {X}\subset \mathbb {R}^{p\times q}$ be a compact set. Then:

- $$\tfrac{1}{2}{{\,\textrm{diam}\,}}\mathcal {X}\le {{\,\textrm{rad}\,}}\mathcal {X}\le {{\,\textrm{diam}\,}}\mathcal {X}$$.
- ${{\,\textrm{cent}\,}}\mathcal {X}$ is compact and convex with no interior points.
- For every $X_0\in \mathbb {R}^{p\times q}$ we have ${{\,\textrm{diam}\,}}(X_0+\mathcal {X})={{\,\textrm{diam}\,}}\mathcal {X}$, ${{\,\textrm{rad}\,}}(X_0+\mathcal {X})={{\,\textrm{rad}\,}}\mathcal {X}$, and ${{\,\textrm{cent}\,}}(X_0+\mathcal {X})=X_0+{{\,\textrm{cent}\,}}\mathcal {X}$.
- If the norm is essentially strictly convex, then the set $\mathcal {X}$ has a unique Chebyshev center.

#### Proof

(a) Observe that from the triangle inequality $\tfrac{1}{2}\left| X_1-X_2\right| \le \tfrac{1}{2}\left| C-X_1\right| +\tfrac{1}{2}\left| C-X_2\right|$. Therefore, taking *C* to be a Chebyshev center, we have $\tfrac{1}{2}\left| X_1-X_2\right| \le {{\,\textrm{rad}\,}}\mathcal {X}$ for all $X_1,X_2\in \mathcal {X}$, which implies $\tfrac{1}{2}{{\,\textrm{diam}\,}}\mathcal {X}\le {{\,\textrm{rad}\,}}\mathcal {X}$. The second inequality is obvious from the definitions (1) and (2).

(b) To prove the boundedness of ${{\,\textrm{cent}\,}}\mathcal {X}$, we note that for any $C\in {{\,\textrm{cent}\,}}\mathcal {X}$ and $X\in \mathcal {X}$ we have $\left| C\right| \le \left| C-X\right| +\left| X\right| \le {{\,\textrm{rad}\,}}\mathcal {X}+\left| X\right|$. Since $\mathcal {X}$ is bounded, we see that ${{\,\textrm{cent}\,}}\mathcal {X}$ is bounded. To prove closedness, consider a sequence $C_i\in {{\,\textrm{cent}\,}}\mathcal {X}$, $i\in \mathbb {N}$, with $\lim _{i\rightarrow \infty } C_i=C$. Let $X\in \mathcal {X}$. Note that $\left| C-X\right| \le \left| C-C_i\right| +\left| C_i-X\right| \le \left| C-C_i\right| +{{\,\textrm{rad}\,}}\mathcal {X}$. Take the limit as $i\rightarrow \infty$ to conclude that $\left| C-X\right| \le {{\,\textrm{rad}\,}}\mathcal {X}$. Thus, $C\in {{\,\textrm{cent}\,}}\mathcal {X}$ and therefore ${{\,\textrm{cent}\,}}\mathcal {X}$ is closed. To prove convexity, suppose that $C_1,C_2\in {{\,\textrm{cent}\,}}\mathcal {X}$. Then, by the triangle inequality, we have the following for any $\alpha \in [0,1]$:

$$\begin{aligned} \left| \alpha C_1+(1-\alpha )C_2-X\right| \le \alpha \left| C_1-X\right| +(1-\alpha )\left| C_2-X\right| \le {{\,\textrm{rad}\,}}\mathcal {X}. \end{aligned}$$

Therefore, for any $C_1,C_2\in {{\,\textrm{cent}\,}}\mathcal {X}$ and any $\alpha \in [0,1]$ we have $\alpha C_1+(1-\alpha )C_2\in {{\,\textrm{cent}\,}}\mathcal {X}$, which implies ${{\,\textrm{cent}\,}}\mathcal {X}$ is convex. Now, we prove that ${{\,\textrm{cent}\,}}\mathcal {X}$ has no interior points. Assume, on the contrary, that the interior of ${{\,\textrm{cent}\,}}\mathcal {X}$ is nonempty. Let $C_1$ belong to the interior of ${{\,\textrm{cent}\,}}\mathcal {X}$. Let $X\in \mathcal {X}$ be such that $|C_1-X|={{\,\textrm{rad}\,}}\mathcal {X}$. Since the interior of ${{\,\textrm{cent}\,}}\mathcal {X}$ is nonempty, there exists a sufficiently small $\alpha >0$ such that $C_2=C_1+\alpha (C_1-X)\in {{\,\textrm{cent}\,}}\mathcal {X}$. This implies that $|C_2-X|=(\alpha +1)|C_1-X|=(\alpha +1){{\,\textrm{rad}\,}}\mathcal {X}$. Since ${{\,\textrm{rad}\,}}\mathcal {X}>0$, we have $|C_2 - X| > {{\,\textrm{rad}\,}}\mathcal {X}$, meaning that $C_2$ is not a Chebyshev center, thus resulting in a contradiction. Therefore, ${{\,\textrm{cent}\,}}\mathcal {X}$ does not have an interior point.

(c) For the diameter, the proof immediately follows from the fact that $(X_0+\mathcal {X})-(X_0+\mathcal {X})=\mathcal {X}-\mathcal {X}$. For the radius, we have

$$\begin{aligned} {{\,\textrm{rad}\,}}(X_0+\mathcal {X})= \min _{C\in \mathbb {R}^{p\times q}} \max _{X\in \mathcal {X}} \left| C-X-X_0\right| =\min _{C\in \mathbb {R}^{p\times q}+X_0} \max _{X\in \mathcal {X}} \left| C-X\right| , \end{aligned}$$

which is equal to ${{\,\textrm{rad}\,}}\mathcal {X}$ as $\mathbb {R}^{p\times q}+X_0=\mathbb {R}^{p\times q}$. For the set of centers, we have

$$\begin{aligned} \begin{aligned} {{\,\textrm{cent}\,}}(X_0+\mathcal {X})&=\{C:\left| C-X\right| \le {{\,\textrm{rad}\,}}(X_0+\mathcal {X}) \ \text {for all}\ X\in X_0+\mathcal {X}\} \\&= \{C:\left| C-X-X_0\right| \le {{\,\textrm{rad}\,}}\mathcal {X} \ \text {for all}\ X\in \mathcal {X}\}\\&=\{C+X_0:\left| C-X\right| \le {{\,\textrm{rad}\,}}\mathcal {X} \ \text {for all}\ X\in \mathcal {X}\}=X_0+{{\,\textrm{cent}\,}}\mathcal {X}. \end{aligned} \end{aligned}$$

(d) Suppose that $C_1,C_2\in {{\,\textrm{cent}\,}}\mathcal {X}$ and the norm $\left| \ \cdot \ \right|$ is essentially strictly convex. We show that $C_1=C_2$. For this, recall from part (b) that ${{\,\textrm{cent}\,}}\mathcal {X}$ is convex. Thus, we have $\tfrac{1}{2}(C_1+C_2)\in {{\,\textrm{cent}\,}}\mathcal {X}$. Hence, there exists an $X\in \mathcal {X}$ such that

4 $$\begin{aligned} \left| \tfrac{1}{2}(C_1+C_2)-X\right| ={{\,\textrm{rad}\,}}\mathcal {X}. \end{aligned}$$

Moreover, we observe that $\left| C_i-X\right| \le {{\,\textrm{rad}\,}}\mathcal {X}$ for $i=1,2$. Thus, it follows from the triangle inequality that

5 $$\begin{aligned} \left| \tfrac{1}{2}(C_1+C_2)-X\right| \le \tfrac{1}{2}\left| C_1-X\right| +\tfrac{1}{2}\left| C_2-X\right| \le {{\,\textrm{rad}\,}}\mathcal {X}. \end{aligned}$$

From (4) and (5) we have

6 $$\begin{aligned} \left| \tfrac{1}{2}(C_1-X)+\tfrac{1}{2}(C_2-X)\right| =\tfrac{1}{2}\left| C_1-X\right| +\tfrac{1}{2}\left| C_2-X\right| ={{\,\textrm{rad}\,}}\mathcal {X}. \end{aligned}$$

Since we have $\left| C_i-X\right| \le {{\,\textrm{rad}\,}}\mathcal {X}$ for $i=1,2$, it follows from (6) that

7 $$\begin{aligned} \left| C_1-X\right| =\left| C_2-X\right| ={{\,\textrm{rad}\,}}\mathcal {X}. \end{aligned}$$

As the norm is essentially strictly convex, (6) implies that $C_1-X=\alpha (C_2-X)$ for some $\alpha \ge 0$. It follows now from (7) that $\alpha =1$. This implies that $C_1=C_2$. $\square$

**Fig. 1:**
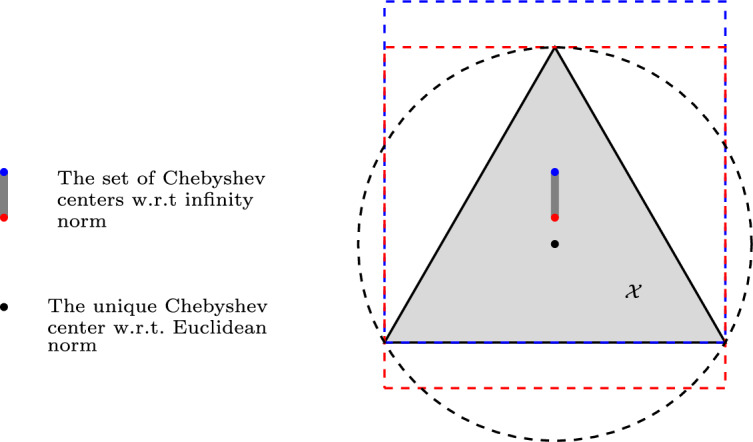
The Chebyshev radius and the set of Chebyshev centers for the closed area defined by an equilateral triangle in terms of Euclidean and infinity norms

As an example, consider the set $\mathcal {X}\subset \mathbb {R}^2$ to be the closed shaded area defined by an equilateral triangle as depicted in Fig. 1. With respect to the Euclidean norm, which is an essentially strictly convex norm, the set of Chebyshev centers is a singleton shown as a black dot. However, with respect to the infinity norm, both points shown in red and blue are Chebyshev centers because they are both centers of a smallest square that contains the triangle. Hence, the set of Chebyshev centers is not a singleton, but the line segment shown in dark gray. This line segment is the center of all squares that contain the triangle and have the same radius as the blue and red ones. The diameter of this set is the length of the triangle’s side for both Euclidean and infinity norms.

### Sets induced by quadratic matrix inequalities

We define

$$\begin{aligned} \pmb {\Pi }_{q,p}\!{:}{=}\! \left\{ \!\begin{bmatrix} \Pi _{11} &  \Pi _{12} \\ \Pi _{21} &  \Pi _{22} \end{bmatrix}\!\in \!\mathbb {S}^{q+p}\!:\! \Pi _{11}\!\in \!\mathbb {S}^q,\Pi _{22}\!\in \!\mathbb {S}^p,\Pi _{22}\le 0,\Pi |\Pi _{22}\ge 0, \ker \Pi _{22}\!\subseteq \!\ker \Pi _{12}\!\right\} \!, \end{aligned}$$

where $\Pi |\Pi _{22}{:}{=}\Pi _{11}-\Pi _{12}\Pi _{22}^\dagger \Pi _{21}$ is the generalized Schur complement of $\Pi$ with respect to $\Pi _{22}$.

For a given $\Pi \in \pmb {\Pi }_{q,p}$, the matrices $\Pi _{11}\in \mathbb {S}^q$, $\Pi _{12}\in \mathbb {R}^{q\times p}$, $\Pi _{21}=\Pi _{12}^\top$, and $\Pi _{22}\in \mathbb {S}^p$ are defined by

$$\begin{aligned} \begin{bmatrix} \Pi _{11} &  \Pi _{12} \\ \Pi _{21} &  \Pi _{22} \end{bmatrix}=\Pi . \end{aligned}$$

We also define

$$\begin{aligned} \pmb {\Pi }_{q,p}^-{:}{=}\{\Pi \in \pmb {\Pi }_{q,p}:\Pi _{22}<0\}. \end{aligned}$$

We define the QMI-induced set $\mathcal {Z}_p(\Pi )$ associated with a matrix $\Pi \in \pmb {\Pi }_{q,p}$ to be

$$\begin{aligned} \mathcal {Z}_p(\Pi ){:}{=}\left\{ Z\in \mathbb {R}^{p\times q}:\begin{bmatrix} I \\ Z \end{bmatrix}^\top \Pi \begin{bmatrix} I \\ Z \end{bmatrix}\ge 0\right\} . \end{aligned}$$

The basic properties of $\mathcal {Z}_p(\Pi )$ are recalled in the following proposition.

#### Proposition 2

( [11, Thms. 3.2 and 3.3]) Let $\Pi \in \pmb {\Pi }_{q,p}$. Then:

- $\mathcal {Z}_p(\Pi )$ is nonempty and convex.
- $\mathcal {Z}_p(\Pi )$ is bounded if and only if $\Pi \in \pmb {\Pi }_{q,p}^-$.
- Suppose that $\Pi \in \pmb {\Pi }_{q,p}^-$. Then, $Z\in \mathcal {Z}_p(\Pi )$ if and only if
  8 $$\begin{aligned} Z=-\Pi _{22}^{-1} \Pi _{21} + (-\Pi _{22})^{-\frac{1}{2}} S (\Pi |\Pi _{22})^{\frac{1}{2}} \end{aligned}$$
  for some $S\in \mathbb {R}^{p\times q}$ satisfying $SS^\top \le I$.

**Fig. 2:**
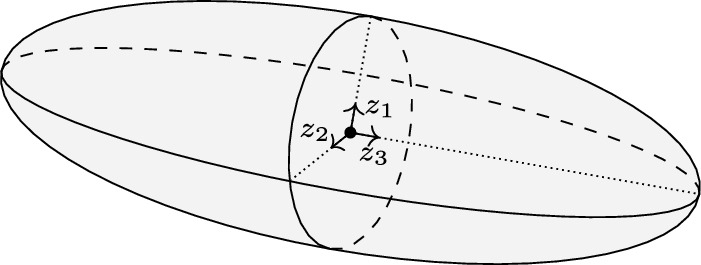
The closed shaded area defined by an ellipsoid is an example of a QMI-induced set

As an example, consider the three-dimensional ellipsoid $\tfrac{z_1^2}{a^2}+\tfrac{z_2^2}{b^2}+\tfrac{z_3^2}{c^2}=1$, depicted in Fig. 2. The region enclosed by this ellipsoid, $\tfrac{z_1^2}{a^2}+\tfrac{z_2^2}{b^2}+\tfrac{z_3^2}{c^2}\le 1$, is a QMI-induced set with $$Z^\top =\begin{bmatrix} z_1&z_2&z_3 \end{bmatrix}$$ and $\Pi$ being a diagonal matrix with diagonal entries of 1, $-\tfrac{1}{a^2}$, $-\tfrac{1}{b^2}$, and $-\tfrac{1}{c^2}$. In this example, (8) is equivalent to the parametrization of the ellipsoid in the spherical coordinates.

### Unitarily invariant norms

In this paper, we focus on unitarily invariant matrix norms.

#### Definition 3

( [37, p. 835]) A matrix norm $\left| \ \cdot \ \right|$ on $\mathbb {C}^{n\times m}$ is called *unitarily invariant* if $\left| UMV\right| =\left| M\right|$ for all $M\in \mathbb {C}^{n\times m}$ and all unitary $U\in \mathbb {C}^{n\times n}$ and $V\in \mathbb {C}^{m\times m}$.

For a matrix $M\in \mathbb {C}^{n\times m}$, its

- Frobenius norm $$\sqrt{{{\,\textrm{tr}\,}}(MM^*)}=\sqrt{\sum _{i=1}^{\min \{n,m\}}\sigma _i^2(M)}$$,
- Schatten *p*-norms $(\sum _{i=1}^{\min \{n,m\}}\sigma _i^p(M))^{1/p}$, $p\ge 1$, and
- Ky Fan *k*-norms $\sum _{i=1}^k\sigma _i(M)$, $k\in [1,\min \{n,m\}]$,

are all unitarily invariant [38, p. 92]. This class of norms has an interesting association with so-called symmetric gauge functions.

#### Definition 4

( [39, p. 51]) A function $g:\mathbb {R}^n\rightarrow \mathbb {R}_+$ is called a *symmetric gauge function* if it satisfies the following properties^3^:

- *g* is a norm,
- $g(Px)=g(x)$ for all permutation matrices $P\in \mathbb {R}^{n\times n}$,
- If $$s_i=\pm 1$$, then $$g\left( \begin{bmatrix} x_1&\cdots&x_n \end{bmatrix}^\top \right) =g\left( \begin{bmatrix} s_1x_1&\cdots&s_nx_n \end{bmatrix}^\top \right) $$.

Having the above definition, it is not surprising that a symmetric gauge function of the vector of singular values of a matrix defines a unitarily invariant norm. Interestingly, von Neumann proved in [41] that the converse is also true.

#### Proposition 5

( [37, Fact 11.9.59]) A norm $\left| \ \cdot \ \right|$ on $\mathbb {C}^{n\times m}$ is unitarily invariant if and only if there exists a symmetric gauge function $g:\mathbb {R}^{\min \{n,m\}}\rightarrow \mathbb {R}_+$ such that $\left| M\right| =g(\sigma (M))$ for all $M\in \mathbb {C}^{n\times m}$.

We note that every unitarily invariant norm is associated with a *unique* symmetric gauge function. The uniqueness is an immediate consequence of the symmetry. As an example, the symmetric gauge functions associated with the spectral norm and the Frobenius norm, respectively, are the infinity and the Euclidean vector norms. For a Schatten *p*-norm, $p\ge 1$, its associated symmetric gauge function is the vector *p*-norm.

We know from Proposition 2(d) that for essentially strictly convex norms, the Chebyshev center of a compact set of matrices is unique. The following result shows that the essential strict convexity of a unitarily invariant norm is equivalent to that of its symmetric gauge function.

#### Proposition 6

( [34, Thm. 3.1]) A unitarily invariant norm is essentially strictly convex if and only if its associated symmetric gauge function is essentially strictly convex.

For instance, based on Proposition 6, the Frobenius norm is essentially strictly convex since the Euclidean norm is essentially strictly convex. Therefore, it follows from Proposition 2(d) that the Chebyshev center of a compact set with respect to the Frobenius norm is unique. However, this is not necessarily the case for the spectral norm.

## Main results

The following theorem is the main result of this paper that provides closed-form expressions for a Chebyshev center, the Chebyshev radius, and the diameter of QMI-induced sets.

### Theorem 7

Let $\Pi \in \pmb {\Pi }_{q,p}^-$. Then, for any matrix norm

$$\begin{aligned} -\Pi _{22}^{-1} \Pi _{21}\in {{\,\textrm{cent}\,}}\mathcal {Z}_{p}(\Pi )\hspace{0.5 cm} \text {and}\hspace{0.5 cm} {{\,\textrm{diam}\,}}\mathcal {Z}_{p}(\Pi )=2{{\,\textrm{rad}\,}}\mathcal {Z}_{p}(\Pi ). \end{aligned}$$

For a unitarily invariant norm with the associated symmetric gauge function *g*, we have

$$\begin{aligned} {{\,\textrm{rad}\,}}\mathcal {Z}_{p}(\Pi )=g(\sigma _{[k]}((-\Pi _{22})^{-\frac{1}{2}})\circ \sigma _{[k]}((\Pi |\Pi _{22})^{\frac{1}{2}})), \end{aligned}$$

where $$k=\min \{p,q\}$$.

Recall from the example in Fig. 1 that the sets of Chebyshev centers for different norms do not necessarily intersect. However, Theorem 7 reveals that for a QMI-induced set $\mathcal {Z}_p(\Pi )$ with $\Pi \in \pmb {\Pi }_{q,p}^-$,

9 $$\begin{aligned} {\hat{Z}}=-\Pi _{22}^{-1} \Pi _{21}, \end{aligned}$$

is a *common* Chebyshev center with respect to all matrix norms. In addition, recall from Proposition 2(d) that for essentially strictly convex norms, e.g., Frobenius norm, the set of Chebyshev centers is a singleton. This implies that (9) is the only common center among all (unitarily invariant) norms.

As an example, consider the ellipsoid $\tfrac{z_1^2}{a^2}+\tfrac{z_2^2}{b^2}+\tfrac{z_3^2}{c^2}\le 1$ shown in Fig. 2. For this example, the Chebyshev radius presented in Theorem 7 with respect to the maximum singular value is equal to the largest semi-axis $\max \{a,b,c\}$, that is the radius of the smallest sphere circumscribing the ellipsoid.

To prove Theorem 7, we need three auxiliary results. The first one deals with an upper bound on the unitarily invariant norm of a product of matrices.

### Lemma 8

Let $L\in \mathbb {R}^{l\times p}$, $S\in \mathbb {R}^{p\times q}$, $R\in \mathbb {R}^{q\times r}$, and $k{:}{=}\min \{l,r\}$. Also, let $g:\mathbb {R}^k\rightarrow \mathbb {R}_+$ be a symmetric gauge function. Then,

10 $$\begin{aligned} g(\sigma _{[k]}(LSR))\le g(\sigma _{[k]}(L)\circ \sigma _{[k]}(S)\circ \sigma _{[k]}(R)). \end{aligned}$$

### Proof

The following majorization inequality holds for the singular values of the product of two matrices $X\in \mathbb {R}^{n_x\times m}$ and $Y\in \mathbb {R}^{m\times n_y}$:

11 $$\begin{aligned} \sigma _{[s]}(XY)\prec _w \sigma _{[s]}(X)\circ \sigma _{[s]}(Y), \end{aligned}$$

for any $s\in \mathbb {N}$. A proof of inequality (11) can be found in [19, p. 342] for square matrices, which can be straightforwardly extended to rectangular matrices by taking care of the dimension of the singular value vectors (see [19, p. 299] for further explanations). Next, to extend inequality (11) to the product of three matrices, we consider the fact that if two vectors $x,y\in \mathbb {R}^n_+$ satisfy $x\prec _w y$, then for any $z\in \mathbb {R}_\downarrow ^n$ we have $x\circ z\prec _w y\circ z$ (see [38, Prob. 11.5.16]). Hence, as $\sigma _{[k]}(R)\in \mathbb {R}_\downarrow ^k$, inequality (11) implies $\sigma _{[k]}(LS)\circ \sigma _{[k]}(R)\prec _w \sigma _{[k]}(L)\circ \sigma _{[k]}(S)\circ \sigma _{[k]}(R)$ by choosing $X=L$ and $Y=S$. Thus, by choosing $X=LS$ and $Y=R$ in (11), we have $\sigma _{[k]}(LSR)\prec _w \sigma _{[k]}(LS)\circ \sigma _{[k]}(R)$. Hence, we obtain

12 $$\begin{aligned} \sigma _{[k]}(LSR)\prec _w \sigma _{[k]}(L)\circ \sigma _{[k]}(S)\circ \sigma _{[k]}(R). \end{aligned}$$

In addition, from Ky Fan’s lemma, for vectors $x,y\in \mathbb {R}^n_+$, we have $g(x)\le g(y)$ for all symmetric gauge functions if and only if $x\prec _w y$ (see [19, Prop. 4.B.6]). Therefore, inequality (12) implies (10) for all symmetric gauge functions. $\square$

The second auxiliary result deals with nonexpansive matrices.

### Lemma 9

Let $$\mathcal {S}{:}{=}\{S\in \mathbb {R}^{n\times m}:SS^\top \le I\}$$. Then, $$\mathcal {S}-\mathcal {S}=2\mathcal {S}$$.

### Proof

Let $Z\in 2\mathcal {S}$. Then, $Z=2X$ for some $X\in \mathcal {S}$. Since $-X\in \mathcal {S}$ and $Z=X-(-X)$, we see that $Z\in \mathcal {S}-\mathcal {S}$. This proves that $2\mathcal {S}\subseteq \mathcal {S}-\mathcal {S}$. For the reverse inclusion, let $Z\in \mathcal {S}-\mathcal {S}$. Then, $Z=X-Y$ where $X,Y\in \mathcal {S}$. Note that

13 $$\begin{aligned} \begin{bmatrix} I &  X \\ X^\top &  I \end{bmatrix}\ge 0,\hspace{0.25 cm}\text {and}\hspace{0.25 cm} \begin{bmatrix} I &  -Y \\ -Y^\top &  I \end{bmatrix}\ge 0. \end{aligned}$$

Adding the matrices in (13) yields

$$\begin{aligned} \begin{bmatrix} 2I &  Z \\ Z^\top &  2I \end{bmatrix}\ge 0, \end{aligned}$$

equivalently, $ZZ^\top \le 4I$. Then, $\tfrac{1}{2}Z\in \mathcal {S}$ and thus $Z\in 2\mathcal {S}$. Therefore, $\mathcal {S}-\mathcal {S}\subseteq 2\mathcal {S}$, which completes the proof. $\square$

The third auxiliary result characterizes the diameter and Chebyshev radius and center for sets obtained from nonexpansive matrices.

### Lemma 10

Let $\mathcal {S}{:}{=}\{S\in \mathbb {R}^{p\times q}:SS^\top \le I\}$, $\mathcal {X}{:}{=}L\mathcal {S}R$ with $L\in \mathbb {R}^{l\times p}$ and $R\in \mathbb {R}^{q\times r}$, and $k{:}{=}\min \{r,l\}$. The following statements hold:

- For any norm, we have $0\in {{\,\textrm{cent}\,}}\mathcal {X}$ and ${{\,\textrm{diam}\,}}\mathcal {X}=2{{\,\textrm{rad}\,}}\mathcal {X}$.
- For a unitarily invariant norm with associated symmetric gauge function *g*, we have ${{\,\textrm{rad}\,}}\mathcal {X}=g(\sigma _{[k]}(L)\circ \sigma _{[k]}(R))$.

### Proof

Let $\rho =\max \{\left| X\right| :X\in \mathcal {X}\}$. It follows from Lemma 9 that $\mathcal {X}-\mathcal {X}=2\mathcal {X}$ and from the definition of the diameter (1) that ${{\,\textrm{diam}\,}}\mathcal {X}=\max \{\left| \Delta \right| :\Delta \in 2\mathcal {X}\}=2\rho$. On the one hand, Proposition 1(a) implies that ${{\,\textrm{rad}\,}}\mathcal {X}\ge \rho$. On the other hand, we have

$$\begin{aligned} {{\,\textrm{rad}\,}}\mathcal {X}=\min _{C_0\in \mathbb {R}^{p\times q}} \max _{X\in \mathcal {X}} \left| C_0-X\right| \le \max _{X\in \mathcal {X}} \left| X\right| =\rho . \end{aligned}$$

Therefore, ${{\,\textrm{rad}\,}}\mathcal {X}=\tfrac{1}{2}{{\,\textrm{diam}\,}}\mathcal {X}=\rho$. Hence, it follows from the definition of the set of Chebyshev centers (3) that $0\in {{\,\textrm{cent}\,}}\mathcal {X}$. This proves part (a). To prove part (b), assume that the norm is unitarily invariant with associated symmetric gauge function *g*. We show that $\rho =g(\sigma _{[k]}(L)\circ \sigma _{[k]}(R))$. To do so, observe that

14 $$\begin{aligned} \left| LSR\right| \le g(\sigma _{[k]}(L)\circ \sigma _{[k]}(S)\circ \sigma _{[k]}(R)), \end{aligned}$$

due to Lemma 8. Since $\sigma _1(S)\le 1$ whenever $SS^\top \le I$, inequality (14) yields

15 $$\begin{aligned} \rho \le g(\sigma _{[k]}(L)\circ \sigma _{[k]}(R)). \end{aligned}$$

Now, we construct an element of $\mathcal {X}$ such that its norm is equal the upper bound in (15). For $M\in \{L,R\}$, let $M=U_M\Sigma _M V_M^\top$ be a singular value decomposition where $U_M$ and $V_M$ are orthogonal matrices, and $\Sigma _M$ is the matrix containing zero entries apart from its main diagonal which equals $\sigma (M)$. Moreover, let $\Sigma _S$ be a $p\times q$ matrix of the form $$\begin{bmatrix} I_k &  0 \\ 0 &  0 \end{bmatrix}$$. Note that $S=V_L\Sigma _S U_R^\top$ satisfies $SS^\top \le I$. Therefore, we have $LSR\in \mathcal {X}$ and

$$\begin{aligned} \left| LSR\right| =\left| \Sigma _L\Sigma _S\Sigma _R\right| =g(\sigma _{[k]}(L)\circ \sigma _{[k]}(R)). \end{aligned}$$

Together with (15) this proves that $\rho = g(\sigma _{[k]}(L)\circ \sigma _{[k]}(R))$. $\square$

It is now straightforward to prove Theorem 7 as follows:

### Proof of Theorem 7

From Proposition 2(c), we have

$$\begin{aligned} \mathcal {Z}_{p}(\Pi )=X_0+\mathcal {X} \end{aligned}$$

where $\mathcal {X}=\{LSR:SS^\top \le I\}$, $X_0=-\Pi _{22}^{-1} \Pi _{21}$, $L=(-\Pi _{22})^{-\frac{1}{2}}$, and $R=(\Pi |\Pi _{22})^{\frac{1}{2}}$. Based on Proposition 1(c), we have ${{\,\textrm{diam}\,}}\mathcal {Z}_p(\Pi )={{\,\textrm{diam}\,}}\mathcal {X}$, ${{\,\textrm{rad}\,}}\mathcal {Z}_p(\Pi )={{\,\textrm{rad}\,}}\mathcal {X}$, and ${{\,\textrm{cent}\,}}\mathcal {Z}_p(\Pi )=X_0+{{\,\textrm{cent}\,}}\mathcal {X}$. Therefore, Theorem 7 now follows from Lemma 10. $\square$

**Fig. 3:**
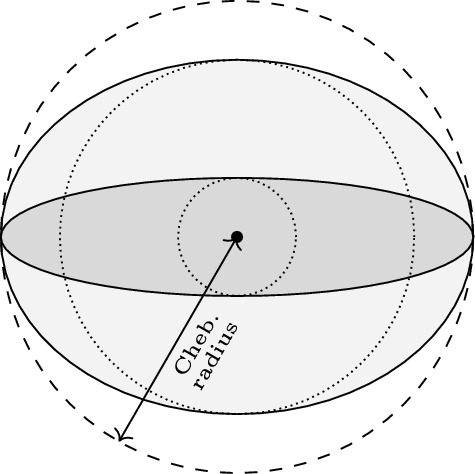
Two ellipses, shown by the dark and light gray areas, with the same Chebyshev radius but different volumes

The Chebyshev centers and radii represent the best outer approximation for a set. However, two sets with the same Chebyshev radius may have different *volumes*, e.g., see Fig. 3. This motivates the study of the radius of a largest ball within the set as an inner approximation, which is another relevant concept in the analysis of QMI-induced sets. To formulate this, let the ball centered at $X_0\in \mathbb {R}^{p\times q}$ with radius $\rho \ge 0$ be defined as

$$\begin{aligned} \mathcal {B}(X_0,\rho ){:}{=}\{X:\left| X-X_0\right| \le \rho \}. \end{aligned}$$

Given $\Pi \in \pmb {\Pi }_{q,p}^-$, the Chebyshev radius of $\mathcal {Z}_p(\Pi )$ is the radius of the smallest ball containing the set $\mathcal {Z}_p(\Pi )$, that is,

$$\begin{aligned} {{\,\textrm{rad}\,}}\mathcal {Z}_p(\Pi )=\min \{\rho :\mathcal {Z}_p(\Pi )\subseteq \mathcal {B}({\hat{Z}},\rho )\}, \end{aligned}$$

where ${\hat{Z}}$ is the common Chebyshev center defined in (9). Now, aside from an outer ball with radius $\rho _1$ where $\mathcal {Z}_p(\Pi )\subseteq \mathcal {B}({\hat{Z}},\rho _1)$, one may consider an inner ball with radius $\rho _2$ such that $\mathcal {B}({\hat{Z}},\rho _2)\subseteq \mathcal {Z}_p(\Pi )$. The radius of such an inner ball is zero if the interior of the QMI-induced set in $\mathbb {R}^{p\times q}$ is empty. This is the case if and only if $\Pi |\Pi _{22}$ has a zero eigenvalue (see [11, Thm. 3.2(c)]). However, in that case, one may study the radius of a largest lower-dimensional ball within the set. Hence, we define

$$\begin{aligned} {{\,\textrm{rad}\,}}_\text {in}\mathcal {Z}_p(\Pi ){:}{=}\max \{\rho :\mathcal {B}({\hat{Z}},\rho )\cap {{\,\textrm{aff}\,}}\mathcal {Z}_p(\Pi )\subseteq \mathcal {Z}_p(\Pi )\}, \end{aligned}$$

which is characterized by the following theorem.

### Theorem 11

Let $\Pi \in \pmb {\Pi }_{q,p}^-$. For a unitarily invariant norm with associated symmetric gauge function *g*, we have

$$\begin{aligned} {{\,\textrm{rad}\,}}_{in }\mathcal {Z}_p(\Pi )=\sigma _*((\Pi |\Pi _{22})^{\frac{1}{2}})\sigma _p((-\Pi _{22})^{-\frac{1}{2}})g(e_1). \end{aligned}$$

Unitarily invariant norms are called *normalized* if their associated symmetric gauge function satisfies $g(e_1)=1$ [42]. All Schatten *p*-norms and Ky Fan *k*-norms are normalized. However, in general, this is not the case as one may consider a weighted norm such that $g(e_1)\ne 1$.

**Fig. 4:**
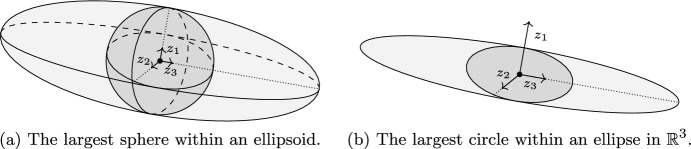
Examples of the inner balls for QMI-induced sets

Once more, consider the example of the ellipsoid $\tfrac{z_1^2}{a^2}+\tfrac{z_2^2}{b^2}+\tfrac{z_3^2}{c^2}\le 1$ shown in Fig. 4a. For this example, the radius of the largest inner ball presented in Theorem 11 with respect to the maximum singular value is equal to the smallest semi-axis $\min \{a,b,c\}$, which is equal to the radius of the largest sphere inscribed in the ellipsoid. Now, consider the example of an ellipse in $\mathbb {R}^3$ shown in Fig. 4b defined by $\tfrac{z_2^2}{b^2}+\tfrac{z_3^2}{c^2}\le 1$ and $z_1=0$. For this example, the radius of the largest sphere within the ellipse is zero. However, the radius of the inner ball presented in Theorem 11 is the radius of the largest circle within the ellipse, which is equal to the smallest semi-axis $\min \{b,c\}$.

To prove Theorem 11, we need the following lemma.

### Lemma 12

Let $\rho \ge 0$, $\mathcal {S}{:}{=}\{S\in \mathbb {R}^{p\times q}:SS^\top \le I\}$, and $\mathcal {X}{:}{=}L\mathcal {S}R$ with $L\in \mathbb {R}^{l\times p}$ and $R\in \mathbb {R}^{q\times r}$. For a unitarily invariant norm with associated symmetric gauge function *g*, we have

16 $$\begin{aligned} \mathcal {B}(0,\rho )\cap {{\,\textrm{aff}\,}}\mathcal {X}\subseteq \mathcal {X} \end{aligned}$$

if and only if $\rho \le \sigma _*(L)\sigma _*(R)g(e_{1})$.

### Proof

It is easy to see that if $L=0$ or $R=0$, then $\mathcal {X}=\{0\}$ is a singleton and the claim holds. Hence, in what follows, we assume that *L* and *R* are both nonzero. For the “if” part, assume that $\rho \le \sigma _*(L)\sigma _*(R)g(e_1)$. Let $X\in \mathcal {B}(0,\rho )\cap {{\,\textrm{aff}\,}}\mathcal {X}$. Since ${{\,\textrm{aff}\,}}\mathcal {X}=\{LTR:T\in \mathbb {R}^{p\times q}\}$, we have $X=\rho LTR$ for some $T\in \mathbb {R}^{p\times q}$ satisfying $\left| LTR\right| \le 1$. Take $S=\rho L^{\dagger }LTRR^{\dagger }$, and observe that

$$\begin{aligned} \sigma _1(S)=\rho \sigma _1(L^{\dagger }LTRR^{\dagger })\le \sigma _*(L)\sigma _*(R)g(e_1) \sigma _1(L^{\dagger })\sigma _1(LTR)\sigma _1(R^{\dagger }). \end{aligned}$$

Since $\sigma _1(L^\dagger )=1/\sigma _*(L)$ and $\sigma _1(R^\dagger )=1/\sigma _*(R)$, we have $\sigma _1(S)\le g(e_1) \sigma _1(LTR)$. Since $\sigma (LTR)\succ _w\sigma _1(LTR)e_1$, it follows from Ky Fan’s lemma [19, Prop. 4.B.6] that

$$\begin{aligned} g(\sigma (LTR))\ge g(\sigma _1(LTR)e_1)=\sigma _1(LTR)g(e_1). \end{aligned}$$

Thus, $\sigma _1(LTR)\le g(\sigma (LTR))/g(e_1)=\left| LTR\right| /g(e_1) \le 1/g(e_1)$. This implies that $\sigma _1(S)\le 1$. Therefore, since $X=LSR$ for some $S\in \mathcal {S}$, we have $X\in \mathcal {X}$. To prove the “only if” part, assume that (16) holds. This implies that for every $T\in \mathbb {R}^{p\times q}$ with $\left| LTR\right| \le 1$ we have $\rho LTR=LSR$ for some $S\in \mathcal {S}$. Based on the singular value decomposition, consider a decomposition of *L* (*R*) as $U_L\Sigma _LV_L^\top$ ($U_R\Sigma _RV_R^\top$) such that $U_L$ and $V_L$ ($U_R$ and $V_R$) are orthogonal matrices and $\Sigma _L$ ($\Sigma _R$) has $\sigma _*(L)$ ($\sigma _*(R)$) on its (1, 1)-th entry. Using this decomposition, we multiply $\rho LTR=LSR$ from left and right by $U_L^\top$ and $V_R$, respectively, to have the equivalent expression

17 $$\begin{aligned} \rho \Sigma _LV_L^\top TU_R\Sigma _R =\Sigma _LV_L^\top S U_R\Sigma _R. \end{aligned}$$

Take $T=V_L\Sigma _T U_R^\top$ where $\Sigma _T$ has all its entries equal to zero but the (1, 1)-entry, which is equal to $1/(\sigma _*(L)\sigma _*(R)g(e_1))$. With this choice, the condition $\left| LTR\right| =1$ is satisfied since

$$\begin{aligned} \left| LTR\right| =\left| \Sigma _L \Sigma _T\Sigma _R\right| =g((1/g(e_1))e_1)=(1/g(e_1))g(e_1)=1. \end{aligned}$$

Substitute $T=V_L\Sigma _T U_R^\top$ in (17) to have

18 $$\begin{aligned} \rho \Sigma _L\Sigma _T\Sigma _R =\Sigma _LV_L^\top S U_R\Sigma _R. \end{aligned}$$

The (1, 1)-th entry of (18) reads $\rho /g(e_1) =\sigma _*(L)\sigma _*(R){\tilde{s}}$, where ${\tilde{s}}$ is the (1, 1)-entry of $V_L^\top S U_R$. Since matrix entries are upper bounded by the largest singular value (see [37, Fact 11.9.23(xii)]), we have ${\tilde{s}}\le \sigma _1(V_L^\top S U_R)=\sigma _1(S)\le 1$. Therefore, we have $\rho /g(e_1) \le \sigma _*(L)\sigma _*(R)$, which completes the proof. $\square$

It is now straightforward to prove Theorem 11 as follows:

### Proof of Theorem 11

From Proposition 2(c) we have $\mathcal {Z}_{p}(\Pi )=X_0+\mathcal {X}$ where $X_0=-\Pi _{22}^{-1} \Pi _{21}$ and $\mathcal {X}=\{LSR:SS^\top \le I\}$ with $L=(-\Pi _{22})^{-\frac{1}{2}}$ and $R=(\Pi |\Pi _{22})^{\frac{1}{2}}$. We also verify that $\mathcal {B}(0,\rho )\cap {{\,\textrm{aff}\,}}\mathcal {X}\subseteq \mathcal {X}$ if and only if $\mathcal {B}(X_0,\rho )\cap {{\,\textrm{aff}\,}}(\mathcal {X}+X_0)\subseteq X_0+\mathcal {X}$. Therefore, Theorem 11 now follows from Lemma 12. $\square$

Using Lemmas 10 and 12, the results of Theorems 7 and 11 can be extended to a more general class of matrix sets, which is presented in the following proposition.

### Proposition 13

Let $\Pi \in \pmb {\Pi }_{q,p}^-$, $\Theta _{L}\in \mathbb {R}^{l\times p}$, and $\Theta _{R}\in \mathbb {R}^{q\times r}$. Then, for any norm we have

19 $$\begin{aligned} -\Theta _L\Pi _{22}^{-1} \Pi _{21}\Theta _R\in {{\,\textrm{cent}\,}}(\Theta _L\mathcal {Z}_{p}(\Pi )\Theta _R), \end{aligned}$$

and

20 $$\begin{aligned} {{\,\textrm{diam}\,}}(\Theta _{L}\mathcal {Z}_{p}(\Pi )\Theta _{R})=2{{\,\textrm{rad}\,}}(\Theta _{L}\mathcal {Z}_{p}(\Pi )\Theta _{R}). \end{aligned}$$

For a unitarily invariant norm with associated symmetric gauge function *g*, we have

21 $$\begin{aligned} {{\,\textrm{rad}\,}}(\Theta _{L}\mathcal {Z}_{p}(\Pi )\Theta _{R})=g(\sigma _{[k]}(\Theta _{L}(-\Pi _{22})^{-\frac{1}{2}})\circ \sigma _{[k]}((\Pi |\Pi _{22})^{\frac{1}{2}}\Theta _{R})), \end{aligned}$$

where $$k{:}{=}\min \{l,r\}$$, and

22 $$\begin{aligned} {{\,\textrm{rad}\,}}_in (\Theta _{L}\mathcal {Z}_{p}(\Pi )\Theta _{R})=\sigma _{*}(\Theta _{L}(-\Pi _{22})^{-\frac{1}{2}}) \sigma _{*}((\Pi |\Pi _{22})^{\frac{1}{2}}\Theta _{R})g(e_1). \end{aligned}$$

### Proof

Based on Proposition 2(c), we have

$$\begin{aligned} \Theta _{L}\mathcal {Z}_{p}(\Pi )\Theta _{R}=X_0+\mathcal {X}, \end{aligned}$$

where $X_0=-\Theta _{L}\Pi _{22}^{-1} \Pi _{21}\Theta _{R}$ and $\mathcal {X}=L\mathcal {S}R$ with $L=\Theta _{L}(-\Pi _{22})^{-\frac{1}{2}}$ and $R=(\Pi |\Pi _{22})^{\frac{1}{2}}\Theta _{R}$. Now, (19), (20), and (21) follow from Lemma 10. Using a similar argument as in the proof of Theorem 11, the proof of (22) follows from Lemma 12. $\square$

## Applications to data-driven modeling and control

In this section, we discuss the applications of the presented results in data-driven modeling and control using a set-membership approach. Consider multiple-input multiple-output (MIMO) autoregressive models with exogenous input (ARX models) given by

23 $$\begin{aligned} y(t+L)=\sum _{i=0}^{L-1}P_iy(t+i)+\sum _{j=0}^{M}Q_ju(t+j)+w(t), \end{aligned}$$

where $y(t)\in \mathbb {R}^p$ is the output, $u(t)\in \mathbb {R}^m$ is the input, $w(t)\in \mathbb {R}^p$ is the process noise, the integers *L* and *M* are known, and matrices $P_i\in \mathbb {R}^{p\times p}$, $i=0,\ldots ,L-1$, and $Q_j\in \mathbb {R}^{p\times m}$, $j=0,\ldots ,M$, are unknowns. We assume that the system is causal, i.e., $M\le L$. Note that input-state models can be captured by choosing $L=1$ and $M=0$. We refer to (23) as the true system and we define

$$\begin{aligned} P_true {:}{=}\begin{bmatrix} P_0&\cdots&P_{L-1} \end{bmatrix}\ \ \text {and}\ \ Q_true {:}{=}\begin{bmatrix} Q_0&\cdots&Q_{M} \end{bmatrix}. \end{aligned}$$

We collect the input–output data

$$\begin{aligned} u(0), \ldots , u(T+M-L),\hspace{0.5 cm} y(0), \ldots , y(T), \end{aligned}$$

from (23), where $T\ge L$. The process noise *w*(*t*) that affects the dynamics during this experiment is unknown, but the matrix

$$\begin{aligned} W_-{:}{=}\begin{bmatrix} w(0)&\cdots&w(T-L) \end{bmatrix} \end{aligned}$$

is assumed to satisfy an energy bound

24 $$\begin{aligned} \begin{bmatrix} I \\ W_-^\top \end{bmatrix}^\top \begin{bmatrix} \Phi _{11} &  \Phi _{12} \\ \Phi _{12}^\top &  \Phi _{22} \end{bmatrix} \begin{bmatrix} I \\ W_-^\top \end{bmatrix}\ge 0, \end{aligned}$$

i.e., $W_-^\top \in \mathcal {Z}_{T-L+1}(\Phi )$, for a given matrix $\Phi \in \pmb {\Pi }_{p,T-L+1}^-$. The reader can refer to [11, p. 4] for the special cases that can be captured by this noise model.

Define the matrices

$$\begin{aligned} \begin{aligned} U_-&{:}{=}\begin{bmatrix} u(0) &  u(1) &  \cdots &  u(T-L) \\ \vdots &  \vdots &  \ddots &  \vdots \\ u(M) &  u(M+1) &  \cdots &  u(T+M-L) \end{bmatrix}, \\ Y_-&{:}{=}\begin{bmatrix} y(0) &  y(1) &  \cdots &  y(T-L) \\ \vdots &  \vdots &  \ddots &  \vdots \\ y(L-1) &  y(L) &  \cdots &  y(T-1) \end{bmatrix},\ \text {and} \\ Y_+&{:}{=}\begin{bmatrix} y(L)&y(L+1)&\cdots&y(T)\end{bmatrix}. \end{aligned} \end{aligned}$$

A pair of real matrices (*P*, *Q*) satisfying

$$\begin{aligned} Y_+=PY_-+QU_-+W_- \end{aligned}$$

for some $W_-^\top \in \mathcal {Z}_{T-L+1}(\Phi )$ is called a *data-consistent system*. We define the set of all data-consistent systems as

$$\begin{aligned} \Sigma {:}{=}\left\{ (P,Q):(Y_+-PY_--QU_-)^\top \in \mathcal {Z}_{T-L+1}(\Phi )\right\} , \end{aligned}$$

which is the set of all (*P*, *Q*) that could have generated the available data for some noise sequence agreeing with (24).

One can verify that the set of data-consistent systems is equivalent to a QMI-induced set (cf. [7, Lem. 4] and [43, Sec. IV]), that is,

25 $$\begin{aligned} (P,Q)\in \Sigma \ if and only if \ \begin{bmatrix} P&Q \end{bmatrix}^\top \in \mathcal {Z}_{s}(N), \end{aligned}$$

where $s{:}{=}Lp+(M+1)m$ denotes the number of columns of $$\begin{bmatrix} Y_-^\top&U_-^\top \end{bmatrix}$$ and

26 $$\begin{aligned} N{:}{=}\begin{bmatrix} I &  Y_+ \\ 0 &  -Y_- \\ 0 &  -U_- \end{bmatrix}\begin{bmatrix} \Phi _{11} &  \Phi _{12} \\ \Phi _{12}^\top &  \Phi _{22} \end{bmatrix}\begin{bmatrix} I &  Y_+ \\ 0 &  -Y_- \\ 0 &  -U_- \end{bmatrix}^\top . \end{aligned}$$

Furthermore, it can be observed that matrix *N* in (26) satisfies $N\in \pmb {\Pi }_{p,s}$. Hence, based on Proposition 2, we have the following facts.

### Proposition 14

The following statements hold:

- $$\Sigma $$ is nonempty.
- $\Sigma$ is bounded if and only if $${{\,\textrm{rank}\,}}\begin{bmatrix} Y_-^\top&U_-^\top \end{bmatrix}=s$$.
- Assume that $\Sigma$ is bounded. Then, $\Sigma$ is a singleton if and only if $N|N_{22}=0$. In this case, $\Sigma =\{(P_{true },Q_{true })\}$ and $$\begin{bmatrix} P_{true }&Q_{true } \end{bmatrix}=-N_{12}N_{22}^{-1}$$.

In the noiseless case, $\Phi _{11}=0$, $\Phi _{12}=\Phi _{21}^\top =0$, and $\Phi _{22}=-I$, the condition of Proposition 14(c), $N|N_{22}=0$, is always satisfied. In the presence of noise, a necessary condition for unique identification is that the noise sequence is such that the left-hand side of (24) is equal to zero, i.e., the noise is of bound-exploring type (cf. [44, Ass. 5]). However, in general, $\mathcal {Z}_{s}(N)$ is not a singleton and $(P_{true },Q_{true })$ cannot be uniquely recovered from the data. Nevertheless, in this scenario, we can still formalize system identification as the problem of finding a Chebyshev center, the Chebyshev radius, and the diameter of $\mathcal {Z}_{s}(N)$.

### Best worst-case estimation

Let $({\hat{P}},{\hat{Q}})$ be an estimation for the true system. The worst-case error of this estimation with respect to a matrix norm $\left| \ \cdot \ \right|$ is defined as

$$\begin{aligned} \max _{(P,Q)\in \Sigma }\left| \begin{bmatrix} {\hat{P}}&{\hat{Q}} \end{bmatrix}-\begin{bmatrix} P&Q \end{bmatrix}\right| . \end{aligned}$$

A best worst-case estimation for the true system is the one for which the worst-case error is minimized. Based on Theorem 7, if the data satisfy $${{\,\textrm{rank}\,}}\begin{bmatrix} Y_-^\top&U_-^\top \end{bmatrix}=s$$, then such an estimation is given by the following Chebyshev center:

27 $$\begin{aligned} \begin{bmatrix} {\hat{P}}&{\hat{Q}} \end{bmatrix}{:}{=}-N_{12}N_{22}^{-1} =\begin{bmatrix} I \\ Y_+^\top \end{bmatrix}^\top \begin{bmatrix} \Phi _{12} \\ \Phi _{22} \end{bmatrix}\begin{bmatrix} Y_- \\ U_- \end{bmatrix}^\top \left( \begin{bmatrix} Y_- \\ U_- \end{bmatrix}\Phi _{22}\begin{bmatrix} Y_- \\ U_- \end{bmatrix}^\top \right) ^{-1}. \end{aligned}$$

We note that (27) is a data-consistent system, $({\hat{P}},{\hat{Q}})\in \Sigma$, and the only common center for all matrix norms. Therefore, $({\hat{P}},{\hat{Q}})$ given by (27) is the *only* estimation that minimizes the worst-case error with respect to *all* matrix norms. For a unitarily invariant norm associated with a symmetric gauge function *g*, the worst-case error of this estimation is

28 $$\begin{aligned} {{\,\textrm{rad}\,}}\mathcal {Z}_s(N)=g(\sigma _{[p]}((-N_{22})^{-\frac{1}{2}})\circ \sigma ((N|N_{22})^{\frac{1}{2}})). \end{aligned}$$

For this estimation, assuming that the norm is normalized, the radius of the largest inner ball is

29 $$\begin{aligned} {{\,\textrm{rad}\,}}_{in }\mathcal {Z}_s(N)=\sigma _*((N|N_{22})^{\frac{1}{2}})\sigma _s((-N_{22})^{-\frac{1}{2}}). \end{aligned}$$

### Least-squares solution as a Chebyshev center

It turns out that the common Chebyshev center (27) coincides with the solution of a matrix least-squares problem. Here, we denote the Frobenius norm by $\left| \ \cdot \ \right| _F$.

#### Proposition 15

Suppose that $${{\,\textrm{rank}\,}}\begin{bmatrix} Y_-^\top&U_-^\top \end{bmatrix}=s$$. Then, we have

30 $$\begin{aligned} ({\hat{P}},{\hat{Q}})=\arg \min _{(P,Q)} \left| (PY_-+QU_--Y_+-\Phi _{12}\Phi _{22}^{-1}) (-\Phi _{22})^{\frac{1}{2}}\right| _{F }. \end{aligned}$$

#### Proof

For given $A\in \mathbb {R}^{s\times l}$ of full row rank and $B\in \mathbb {R}^{p\times l}$, we recall that the least-squares problem

$$\begin{aligned} \arg \min _{X\in \mathbb {R}^{p\times s}} |XA-B|_\text {F} \end{aligned}$$

has a unique solution ${\hat{X}}=BA^\dagger =BA^\top (AA^\top )^{-1}$, see [45, Eq. (12.12)]. Now, to show that (30) holds, we take

$$\begin{aligned} X=\begin{bmatrix} P&Q \end{bmatrix},\ A=\begin{bmatrix} Y_- \\ U_- \end{bmatrix}(-\Phi _{22})^{\frac{1}{2}},\ \text {and}\ B=(Y_++\Phi _{12}\Phi _{22}^{-1})(-\Phi _{22})^{\frac{1}{2}}. \end{aligned}$$

$$\square $$

We note that (30) can be seen as a generalized least-squares problem with the bias term $\Phi _{12}\Phi _{22}^{-1}$ taken into account and $\Phi _{22}$ acting as the precision matrix. If $\Phi _{22}=-I$ and $\Phi _{12}=0$, then (30) is the solution to the ordinary least-squares problem (cf. [20, 46, 47]).

### Prior knowledge

The set of data-consistent systems $\Sigma$ is merely constructed based on the collected data and the known model class. However, incorporating further prior knowledge may result in a smaller estimation error. Let us introduce the set $\Sigma _{pk }$ representing any prior knowledge of the true system coefficients. Given $\Sigma$ and $\Sigma _{pk }$, the set of data-consistent systems complying with the prior knowledge is $\Sigma \cap \Sigma _{pk }$. In the case that $\Sigma \cap \Sigma _{pk }$ is bounded and QMI-induced, the result of Theorem 7 can be extended accordingly. Note that prior knowledge could also reduce the rank condition on the data matrix $$\begin{bmatrix} Y_-^\top&U_-^\top \end{bmatrix}$$ as $\Sigma \cap \Sigma _{pk }$ may be bounded even if $\Sigma$ is unbounded (see Proposition 14(b)).

As an example, suppose that for known matrices $\Omega _2\in \mathbb {R}^{h\times s}$ and $\Omega _1\in \mathbb {R}^{p\times s}$ we have

31 $$\begin{aligned} \Sigma _{pk }{:}{=}\left\{ (P,Q):\begin{bmatrix} P&Q \end{bmatrix}=H\Omega _2+\Omega _1\ \text {for some}\ H\in \mathbb {R}^{p\times h}\right\} . \end{aligned}$$

This type of constraint can be used when some columns of $$\begin{bmatrix} P_true&Q_true \end{bmatrix}$$ are known a priori so that the unknown terms are included in *H*. It follows now from (25) and (31) that $(P,Q)\in \Sigma \cap \Sigma _{pk }$ is equivalent to $(H\Omega _2+\Omega _1)^\top \in \mathcal {Z}_s(N)$. This inclusion can be written as

32 $$\begin{aligned} H^\top \in \mathcal {Z}_{h}(N_{pk }) \end{aligned}$$

with $N_{pk }$ defined as

$$\begin{aligned} N_{pk }{:}{=}\begin{bmatrix} I &  \Omega _1 \\ 0 &  \Omega _2 \end{bmatrix}N \begin{bmatrix} I &  \Omega _1 \\ 0 &  \Omega _2 \end{bmatrix}^\top . \end{aligned}$$

Therefore, $(P,Q)\in \Sigma \cap \Sigma _{pk }$ if and only if (32) holds. In this case, one can observe that for $\Sigma \cap \Sigma _{pk }$ to be bounded, we need $$\begin{bmatrix} Y_-^\top&U_-^\top \end{bmatrix}\Omega _2^\top $$ to be full column rank (cf. Proposition 14(b)). Having a bounded $\Sigma \cap \Sigma _{pk }$, the results of Theorem 7 hold for the set of data-consistent systems incorporating prior knowledge (31) by replacing *N* with $N_{pk }$.

### The choice of norm

Theorem 7 is applicable to arbitrary unitarily invariant norms. Depending on the application, certain norms could be more relevant than others. For instance, suppose that the goal is to design a robust controller for the set of data-consistent systems. In this case, considering a Chebyshev center $({\hat{P}},{\hat{Q}})$ to be the nominal system with the bound on the uncertainty $(\Delta _P,\Delta _Q)$ given by the Chebyshev radius with respect to the spectral norm, one may check the feasibility to design a robust controller for all systems in the form of $({\hat{P}}+\Delta _P,{\hat{Q}}+\Delta _Q)$. This can be accomplished, e.g., using the results in [30]. Such a parametrization can be obtained from the following proposition. Here, $\lambda _min (M)$ and $\lambda _max (M)$ denote the smallest and largest eigenvalues of a square matrix *M*, respectively

#### Proposition 16

Suppose that $N_{22}<0$. Then, we have

$$\begin{aligned} \sigma _{1}\left( \begin{bmatrix} \Delta _P&\Delta _Q \end{bmatrix}\right) \le \rho , \end{aligned}$$

for all $(\Delta _P,\Delta _Q)\in \Sigma -({\hat{P}},{\hat{Q}})$ if and only if

$$\begin{aligned} \rho \ge \sqrt{\frac{\lambda _{max }(N|N_{22})}{\lambda _{min }(-N_{22})}}. \end{aligned}$$

#### Proof

It suffices to show that, with respect to the spectral norm, we have

33 $$\begin{aligned} {{\,\textrm{rad}\,}}\mathcal {Z}_s(N)=\sqrt{\frac{\lambda _{max }(N|N_{22})}{\lambda _{min }(-N_{22})}}. \end{aligned}$$

Recall that the symmetric gauge function associated with the spectral norm is the infinity norm on $\mathbb {R}^n$. Thus, it follows from (28) that

34 $$\begin{aligned} {{\,\textrm{rad}\,}}\mathcal {Z}_s(N)=\sigma _{1}\left( \left( -N_{22})^{-\frac{1}{2}}\right) \sigma _1\left( (N|N_{22})^{\frac{1}{2}}\right) \right) . \end{aligned}$$

Since $N|N_{22}\ge 0$ and $N_{22}<0$, we have

35 $$\begin{aligned} \begin{aligned} \sigma _1\left( (N|N_{22})^{\frac{1}{2}}\right)&=\lambda _\text {max}\left( (N|N_{22})^{\frac{1}{2}}\right) =\sqrt{\lambda _\text {max}(N|N_{22})}\ \text { and}\\ \sigma _{1}\left( (-N_{22})^{-\frac{1}{2}}\right)&=\lambda _\text {max}\left( (-N_{22})^{-\frac{1}{2}}\right) =\sqrt{\lambda _\text {max}\left( (-N_{22})^{-1}\right) }=\frac{1}{\sqrt{\lambda _\text {min}(-N_{22})}}. \end{aligned} \end{aligned}$$

Now, (33) follows from (34) and (35). $\square$

A similar observation to Proposition 16 was also made in [48]. For the special case $L=1$, $M=0$, $\Phi _{22}=-I$, and $\Phi _{12}=\Phi _{21}^\top =0$, it is proven in [48, Lem. IV.5] that the set of data-consistent systems $\mathcal {Z}_s(N)$ is contained in a ball with radius $\sqrt{\lambda _{max }(N | N_{22}) / \lambda _{min }(-N_{22})}$ centered at $-N_{22}^{-1}N_{21}$. Interestingly, our result shows that this ball is the one with *smallest* radius containing $\mathcal {Z}_s(N)$.

The majority of the literature on robust control deals with uncertainties that are bounded with respect to the spectral norm. Nevertheless, it was argued in [31] that in some cases the use of the Frobenius norm is preferred. We show this by means of an example borrowed from [31]. Suppose that $\Delta _Q=0$ and consider two possibilities for the value of $\Delta _P$:

$$\begin{aligned} \Delta _P^{(1)}=\begin{bmatrix} 1 &  0 \\ 0 &  0 \end{bmatrix}\ \text { and }\ \Delta _P^{(2)}=\begin{bmatrix} 1 &  0 \\ 0 &  1 \end{bmatrix}. \end{aligned}$$

The matrix $\Delta _P^{(2)}$ has one more nonzero entry compared to $\Delta _P^{(1)}$, which can be seen as a less favorable uncertainty. However, the spectral norms of both $\Delta _P^{(1)}$ and $\Delta _P^{(2)}$ are equal, $\sigma _1(\Delta _P^{(1)})=\sigma _1(\Delta _P^{(2)})=1$. Thus, the difference between $\Delta _P^{(1)}$ and $\Delta _P^{(2)}$ cannot be inferred directly from their spectral norms. In this case, working with the Frobenius norm might be preferable as it reflects such differences in its value, i.e., $|\Delta _P^{(1)}|_\text {F}=1$ and $|\Delta _P^{(2)}|_\text {F}=2$.

### Experiment design

Provided that input–output data are given, one can obtain an estimation for the true system using (27), and characterize the estimation error using (28) and (29). However, in case there are no data (or the given data do not satisfy the desired worst-case error), one can think of collecting (more) data such that the desired accuracy is achieved. For this, given $\rho >0$ and a norm $\left| \ \cdot \ \right|$, the goal is to find a finite-length input signal by which the data generated by the true system result in ${{\,\textrm{rad}\,}}\mathcal {Z}_s(N) \le \rho$. This is referred to as *experiment design*.

It was shown in [49] that using some prior knowledge of the true system, for a given $\delta >0$, one can find a sequence of inputs such that the data generated by the true system satisfy

$$\begin{aligned} \sigma _{s}\left( \begin{bmatrix} Y_-^\top&U_-^\top \end{bmatrix}\right) \ge \delta . \end{aligned}$$

Here, we show that this result from [49] can be used in experiment design for a desired identification accuracy with respect to arbitrary unitarily invariant norms. To that end, first, we study the relation between $$\sigma _{s}\left( \begin{bmatrix} Y_-^\top&U_-^\top \end{bmatrix}\right) $$ and ${{\,\textrm{rad}\,}}\mathcal {Z}_s(N)$.

#### Proposition 17

Suppose that $$\begin{bmatrix} Y_-^\top&U_-^\top \end{bmatrix}$$ has full column rank. Then, for a unitarily invariant norm with the associated symmetric gauge function *g*, we have

36 $$\begin{aligned} {{\,\textrm{rad}\,}}\mathcal {Z}_s(N)\le \frac{\sqrt{\sigma _1(\Phi |\Phi _{22})}}{\sigma _{s}\left( \begin{bmatrix} Y_-^\top&U_-^\top \end{bmatrix}\right) }g(\textbf{1}), \end{aligned}$$

where $\textbf{1}\in \mathbb {R}^p$ denotes the vector with all entries equal to 1.

#### Proof

We observe that

$$\begin{aligned} N|N_{22}=\Phi |\Phi _{22}+\begin{bmatrix} I \\ Y_+^\top \end{bmatrix}^\top \begin{bmatrix} \Phi _{12} \\ \Phi _{22} \end{bmatrix}\Psi \begin{bmatrix} \Phi _{12} \\ \Phi _{22} \end{bmatrix}^\top \begin{bmatrix} I \\ Y_+^\top \end{bmatrix}, \end{aligned}$$

where

$$\begin{aligned} \Psi =\Phi _{22}^{-1}-\begin{bmatrix} Y_- \\ U_- \end{bmatrix}^\top \left( \begin{bmatrix} Y_- \\ U_- \end{bmatrix}\Phi _{22}\begin{bmatrix} Y_- \\ U_- \end{bmatrix}^\top \right) ^{-1}\begin{bmatrix} Y_- \\ U_- \end{bmatrix}. \end{aligned}$$

Since $\Phi _{22}<0$, using a Schur complement argument, one can see that $\Psi \le 0$. Thus,

37 $$\begin{aligned} N|N_{22}\le \Phi |\Phi _{22}. \end{aligned}$$

This implies that $\sigma _1(N|N_{22})\le \sigma _1(\Phi |\Phi _{22})$. Therefore, we have

$$\begin{aligned} \sigma (N|N_{22})\prec _w \sigma _1(N|N_{22}) \textbf{1} \prec _w \sigma _1(\Phi |\Phi _{22}) \textbf{1}. \end{aligned}$$

Hence,

$$\begin{aligned} \sigma ((N|N_{22})^{\frac{1}{2}})\prec _w \sqrt{\sigma _1(\Phi |\Phi _{22})} \textbf{1}. \end{aligned}$$

We further observe that the largest singular value of $(-N_{22})^{-\frac{1}{2}}$ is equal to $1/\sqrt{\sigma _s(-N_{22})}$. Thus, we have

$$\begin{aligned} \sigma _{[p]}\left( (-N_{22})^{-\frac{1}{2}}\right) \prec _w \frac{1}{\sqrt{\sigma _s(-N_{22})}} \textbf{1}. \end{aligned}$$

Therefore,

$$\begin{aligned} \sigma _{[p]}\left( (-N_{22})^{-\frac{1}{2}}\right) \circ \sigma \left( (N|N_{22})^{\frac{1}{2}}\right) \prec _w \sqrt{\frac{\sigma _1(\Phi |\Phi _{22})}{\sigma _s(-N_{22})}} \textbf{1}. \end{aligned}$$

Now, it follows from (28) and Ky Fan’s lemma [19, Prop. 4.B.6] that

$$\begin{aligned} {{\,\textrm{rad}\,}}\mathcal {Z}_s(N)\le \sqrt{\frac{\sigma _1(\Phi |\Phi _{22})}{\sigma _s(-N_{22})}}g(\textbf{1}). \end{aligned}$$

This, together with $$\sigma _{s}\left( \begin{bmatrix} Y_-^\top&U_-^\top \end{bmatrix}\right) =\sqrt{\sigma _s(-N_{22})}$$, completes the proof. $\square$

As an example, suppose that the norm is the spectral norm. In addition, suppose that the noise model is given by $\Phi _{11}=\varepsilon ^2 I$, $\Phi _{12}=0$, and $\Phi _{22}=-I$. In that case, we have $g(\textbf{1})=1$ and $\sigma _1(\Phi |\Phi _{22})=\varepsilon ^2$. It follows now from (36) that

$$\begin{aligned} {{\,\textrm{rad}\,}}\mathcal {Z}_s(N)\le \frac{\varepsilon }{\sigma _{s}\left( \begin{bmatrix} Y_-^\top&U_-^\top \end{bmatrix}\right) }. \end{aligned}$$

For this particular case, a similar observation was also made in [49, Ex. 1].

Inequality (36) represents the effect of a *signal-to-noise ratio* on the identification accuracy with respect to an arbitrary unitarily invariant norm. It follows now from this result that, in the presence of noise, the desired identification accuracy ${{\,\textrm{rad}\,}}\mathcal {Z}_s(N)\le \rho$ is met if the data satisfy

$$\begin{aligned} \sigma _{s}\left( \begin{bmatrix} Y_-^\top&U_-^\top \end{bmatrix}\right) \ge \frac{\sqrt{\sigma _1(\Phi |\Phi _{22})}}{\rho }g(\textbf{1}). \end{aligned}$$

Having this relation, one can use [49, Thm. 6] to design an experiment for identification with a desired accuracy. This result quantifies how large $$\sigma _{s}\left( \begin{bmatrix} Y_-^\top&U_-^\top \end{bmatrix}\right) $$ should be in order to achieve a desired identification accuracy.

The role of the smallest singular value of the data matrix $$\begin{bmatrix} Y_-^\top&U_-^\top \end{bmatrix}$$ in the identification accuracy is revealed in Proposition 17. Now, the following proposition shows that the largest singular value of this matrix also contributes to the volume of the set of data-consistent systems by affecting the radius of the largest inner ball.

#### Proposition 18

Suppose that $$\begin{bmatrix} Y_-^\top&U_-^\top \end{bmatrix}$$ has full column rank and the norm is unitarily invariant and normalized. Then, we have

38 $$\begin{aligned} {{\,\textrm{rad}\,}}_in \mathcal {Z}_s(N)\le \frac{\sqrt{\sigma _1(\Phi |\Phi _{22})}}{\sigma _{1}\left( \begin{bmatrix} Y_-^\top&U_-^\top \end{bmatrix}\right) }. \end{aligned}$$

#### Proof

It follows from (37) that $\sigma _*(N|N_{22})\le \sigma _1(N|N_{22})\le \sigma _1(\Phi |\Phi _{22})$. We also observe that

$$\begin{aligned} \sigma _s\left( (-N_{22})^{-\frac{1}{2}}\right) =\frac{1}{\sigma _1\left( (-N_{22})^{\frac{1}{2}}\right) }=\frac{1}{\sigma _{1}\left( \begin{bmatrix} Y_-^\top&U_-^\top \end{bmatrix}\right) }. \end{aligned}$$

Substituting this into (29) yields (38). $\square$

### Examples

In what follows, we consider two examples^4^. The first one is a single-input single-output (SISO) system where the set of data-consistent systems is an ellipsoid in $\mathbb {R}^4$ allowing for a better demonstration of the introduced notions. The second example is a MIMO system, by which the effect of signal-to-noise ratio (SNR) on the Chebyshev radii with respect to several norms is shown through simulations.

#### Mass-spring-damper

**Fig. 5:**
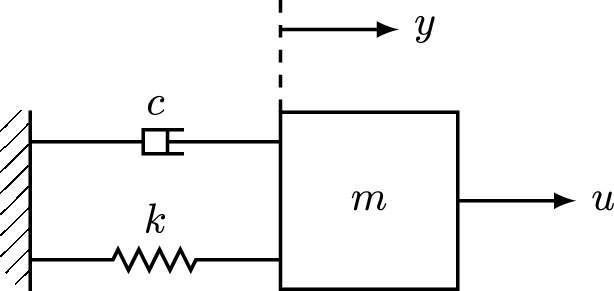
Mass-spring-damper system for Example 1

Consider the model of the mass-spring-damper system in Fig. 5, discretized using the zero-order hold method with a sample time of $1\ s$. Here, *y* denotes the position of the mass with respect to the equilibrium, and *u* denotes the external force. The true parameters of the system are $m=2\ kg$, $k=3\ N/m$, and $c=1\ Ns/m$. The mass position *y*(*t*) is measured through a noisy experiment during a time horizon of 5 seconds. For such a system, we consider the following model:

$$\begin{aligned} y(t+2)=P_0 y(t)+P_1 y(t+1)+Q_0 u(t) +Q_1 u(t+1)+w(t). \end{aligned}$$

The true system coefficients are $P_0=-0.6065$, $P_1=0.5659$, $Q_0=0.1583$, and $Q_1=0.1886$. The process noise is assumed to satisfy the energy bound $\sum _{t=0}^3(w(t)-0.005)^2\le 10^{-4}$ that can be captured by (24), $W_-^\top \in \mathcal {Z}_4(\Phi )$ and $\Phi \in \pmb {\Pi }_{1,4}^-$, with $\Phi _{11}=0$, $\Phi _{22}=-I_4$, and $$\Phi _{12}=\Phi _{21}^\top =0.005\begin{bmatrix} 1&1&1&1 \end{bmatrix}$$.

During the experiment, starting from $y(0)=0$, $y(1)=1$, we apply the input sequence $u(0)=1$, $u(2)=0$, $u(3)=-1$, $u(4)=0$, and $u(5)=1$, which makes $U_-$ equal to

$$\begin{aligned} U_-=\begin{bmatrix} 1 &  0 &  -1 &  0 \\ 0 &  -1 &  0 &  1 \\ \end{bmatrix}. \end{aligned}$$

Now, consider a simulation with the noise sequence

$$\begin{aligned} W_-=\begin{bmatrix} 0.0105&-0.0013&0.0092&0.0084 \end{bmatrix}, \end{aligned}$$

for which the collected output data are

$$\begin{aligned} \begin{aligned} Y_-&=\begin{bmatrix} 0 &  1 &  0.7347 &  -0.3807 \\ 1 &  0.7347 &  -0.3807 &  -0.8101 \end{bmatrix}, \\ Y_+&=\begin{bmatrix} 0.7347&-0.3807&-0.8101&-0.0305 \end{bmatrix}. \end{aligned} \end{aligned}$$

**Fig. 6:**
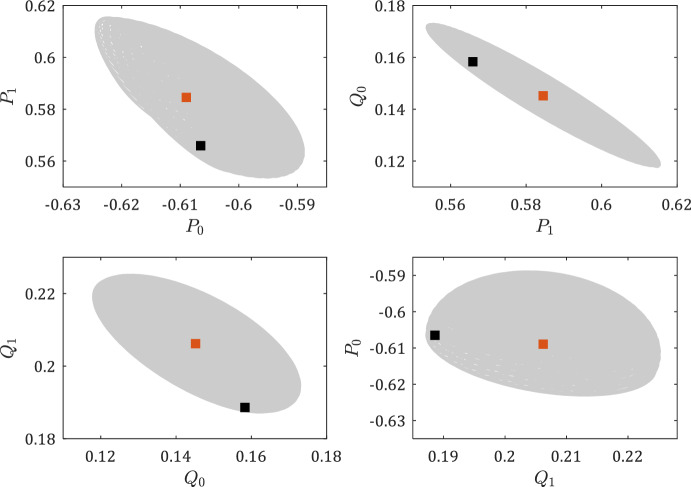
The set of data-consistent systems is shown by the gray area. The red and black squares show the Chebyshev center and the true system, respectively

For this case, the Chebyshev center (27) is

$$\begin{aligned} {\hat{P}}=\begin{bmatrix} -0.6065&0.5659 \end{bmatrix},\hspace{0.25cm} {\hat{Q}}=\begin{bmatrix} 0.1583&0.1886 \end{bmatrix}, \end{aligned}$$

and the Chebyshev radius in terms of the Euclidean norm is ${{\,\textrm{rad}\,}}\mathcal {Z}_4(N)=0.0458$. For the described simulation, the set of data-consistent systems, the true system, and the Chebyshev center (27) are shown in Fig. 6.

**Fig. 7:**
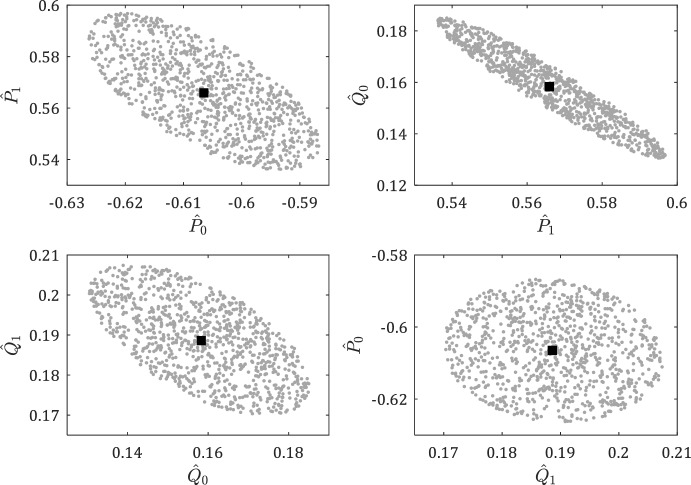
The gray points show the Chebyshev centers obtained for different sequences of the noise. The black square shows the true system

To visualize the results beyond a single simulation, we obtain 1000 simulations for uniformly distributed random sequences of noise normalized to comply with $W_-^\top \in \mathcal {Z}_4(\Phi )$. The Chebyshev centers (27) are obtained for each simulation and are shown by the gray points in Fig. 7.

#### Resistor-capacitor circuit

**Fig. 8:**
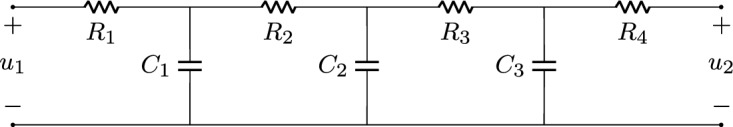
RC circuit for Example 2

Consider the model of the resistor-capacitor (RC) circuit in Fig. 8, discretized using the zero-order hold method with a sample time of $1\ s$. Here, the output variables $y_1$, $y_2$, and $y_3$ are the voltages across the capacitors $C_1$, $C_2$, and $C_3$, respectively, and the input variables $u_1$ and $u_2$ are the voltage sources. The true parameters of the system are $R_1=7\ \Omega$, $R_2=5\ \Omega$, $R_3=10\ \Omega$, $R_4=15\ \Omega$, $C_1=0.5\ F$, $C_2=0.4\ F$, and $C_3=0.6\ F$. We collect the input–output data for a duration of 10 seconds, starting from $y(0)=0$, where the noise sequence is assumed to satisfy $W_-^\top \in \mathcal {Z}_{10}(\Phi )$ and $\Phi \in \pmb {\Pi }_{3,10}^-$ with $\Phi _{11}=10^{-6} I_3$, $\Phi _{22}=-I_{10}$, and $\Phi _{12}=\Phi _{21}^\top =0$. Let the SNR between the input and the noise be defined as $\sigma _m(U_-)/\sigma _1(W_-)$. We run 1000 simulations per SNR for a range of SNR from 1 to $10^3$. For each simulation, uniformly distributed random sequences of noise and input are generated and then normalized, respectively, to attain the required noise bound $\sigma _1(W_-)\le 10^{-3}$ and the SNR value.

**Fig. 9:**
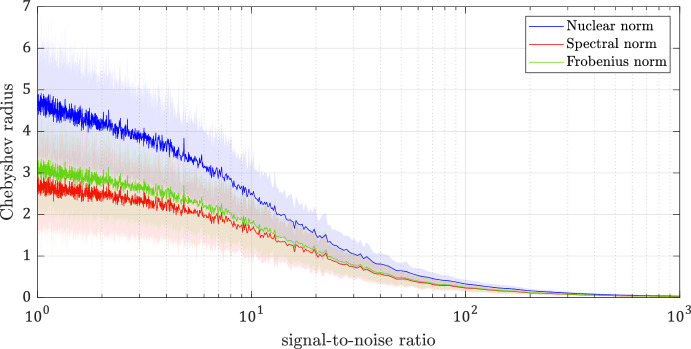
The Chebyshev radii with respect to different norms. The curves indicate the mean values with the shaded area indicating the standard deviation over 1000 simulations per SNR

**Fig. 10:**
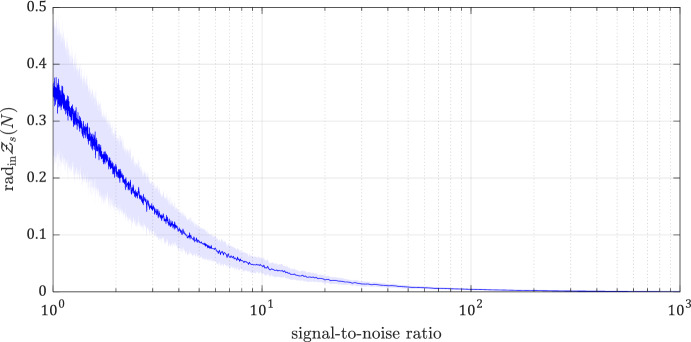
The radius of the largest ball within the set of data-consistent systems. The curves indicate the mean values with the shaded area indicating the standard deviation over 1000 simulations per SNR

Figure 9 shows the Chebyshev radii with respect to the nuclear, spectral, and Frobenius norms. Figure 10 shows the radius of the largest ball within the set of data-consistent systems, ${{\,\textrm{rad}\,}}_in \mathcal {Z}_s(N)$, which has the same value for nuclear, spectral, and Frobenius norms as they are all normalized, i.e., they satisfy $g(e_1)=1$. In both figures, the solid curves are the mean values, and the shaded areas indicate the standard deviation computed through 1000 simulations per SNR. These simulations demonstrate the results of Propositions 17 and 18, where it was shown that both ${{\,\textrm{rad}\,}}\mathcal {Z}_s(N)$ and ${{\,\textrm{rad}\,}}_\text {in} \mathcal {Z}_s(N)$ admit upper bounds in terms of an SNR measure.

## Conclusions

In this work, we have formulated analytic expressions for Chebyshev centers, the Chebyshev radii, and the diameters of certain types of QMI-induced sets with respect to arbitrary unitarily invariant matrix norms. It has been shown that the presented Chebyshev center is the only common center among all matrix norms. We also presented the radius of a largest ball within a QMI-induced set. We discussed the applications of the provided results in data-driven modeling and control of unknown linear time-invariant systems. A notion that has not been fully studied in this work is the *volume* of the QMI-induced sets, which can be a subject for future studies. Moreover, future work could focus on applications of the obtained results in experiment design for system identification with prescribed accuracy.

## Data Availability

No datasets were generated or analyzed during the current study.

## Footnotes

1 For sets that are not compact, one can define the Chebyshev centers and radii by taking the infimum and supremum instead of the maximum and minimum, see [35].

2 Note that Chebyshev centers and radii are defined differently in some references such as [36, Chapter 8.5.2], where the Chebyshev center is referred to as the center of a largest ball that lies within the set.

3 The concept of a gauge function, property (a), is due to Minkowski (see [40, 1.1(d)]). The notion of symmetry, properties (b) and (c), was introduced by von Neumann in [41].

4 The MATLAB codes for the examples of this section can be found at https://github.com/a-shakouri/set-membership-system-identification.
